# Activation of mitophagy leads to decline in Mfn2 and loss of mitochondrial mass in Fuchs endothelial corneal dystrophy

**DOI:** 10.1038/s41598-017-06523-2

**Published:** 2017-07-27

**Authors:** Anne-Sophie Benischke, Shivakumar Vasanth, Takashi Miyai, Kishore Reddy Katikireddy, Tomas White, Yuming Chen, Adna Halilovic, Marianne Price, Francis Price, Paloma B. Liton, Ula V. Jurkunas

**Affiliations:** 1 Schepens Eye Research Institute, Massachusetts Eye and Ear, Harvard Medical School, Boston, MA USA; 2 Price Vision Group, Indianapolis, IN USA; 3 Department of Ophthalmology, Duke University, Durham, NC USA

## Abstract

Human corneal endothelial cells (HCEnCs) are terminally differentiated cells that have limited regenerative potential. The large numbers of mitochondria in HCEnCs are critical for pump and barrier function required for corneal hydration and transparency. Fuchs Endothelial Corneal Dystrophy (FECD) is a highly prevalent late-onset oxidative stress disorder characterized by progressive loss of HCEnCs. We previously reported increased mitochondrial fragmentation and reduced ATP and mtDNA copy number in FECD. Herein, carbonyl cyanide *m*-chlorophenyl hydrazone (CCCP)-induced mitochondrial depolarization decreased mitochondrial mass and Mfn2 levels, which were rescued with mitophagy blocker, bafilomycin, in FECD. Moreover, electron transport chain complex (I, V) decrease in FECD indicated deficient mitochondrial bioenergetics. Transmission electron microscopy of FECD tissues displayed an increased number of autophagic vacuoles containing degenerated and swollen mitochondria with cristolysis. An elevation of LC3-II and LAMP1 and downregulation of Mfn2 in mitochondrial fractions suggested that loss of fusion capacity targets fragmented mitochondria to the pre-autophagic pool and upregulates mitophagy. CCCP-induced mitochondrial fragmentation leads to Mfn2 and LC3 co-localization without activation of proteosome, suggesting a novel Mfn2 degradation pathway via mitophagy. These data indicate constitutive activation of mitophagy results in reduction of mitochondrial mass and abrogates cellular bioenergetics during degeneration of post-mitotic cells of ocular tissue.

## Introduction

Human corneal endothelial cells (HCEnCs) are a monolayer of hexagonal cells situated in the posterior portion of the cornea facing the anterior chamber of the eye^1^. HCEnCs are arrested in post-mitotic state and have minimal proliferative capacity *in vivo* ^1, 2^. The key role of corneal endothelial cells is to maintain corneal hydration via barrier and pump functions, as the deficiency in either function is not compatible with clear vision. Fuchs endothelial corneal dystrophy (FECD) is the most common age-related degeneration of HCEnCs manifesting in corneal edema and blindness and is the major reason for corneal transplantation performed in the United States^3^. FECD is characterized by loss of HCEnC and accumulation of extracellular matrix deposits, termed guttae, which arise from the underlying Descemet’s membrane^4^. Because HCEnCs are arrested in post-mitotic state and minimally proliferative *in vivo*, loss of endothelial cells seen in FECD is considered permanent^2^.

Recent studies have linked oxidative stress with the pathogenesis of FECD^5–9^. Specifically, mitochondrial dysfunction in FECD has been investigated in several studies. Previously, decreased numbers of mitochondria along with decreased activity of cytochrome oxidase, the major respiratory chain enzyme^10^, were detected by immunohistochemistry and a decreased level of mitochondria-encoded respiratory chain transcripts was detected by serial analysis of gene expression^11^. Moreover, decreased mitochondrial antioxidant capacity was detected in FECD due to underexpression of SOD-2 and Prx-5^12^. More recently, studies have shown decreased mitochondrial DNA copy number, mitochondrial DNA damage, and mitochondrial fragmentation in FECD^13^. Since HCEnCs are post–mitotic and exhibit a high rate of metabolism due to habitual pumping of ions^14^, the mitochondrial respiratory chain becomes a major source of reactive oxygen species (ROS) production^15^ and poses a great risk to the mitochondrial biogenesis. When dysfunctional mitochondria are not degraded, they become an even higher source of ROS production and amplify oxidative damage to the cell^16^.

Repetitive cycles of mitochondrial fusion and fission have evolved as protective mechanisms in response to mitochondrial damage and are tightly regulated by mitochondrial quality control systems^17^. During starvation or under conditions of cellular stress, autophagy is activated to remove undesired organelles and cytoplasmic constituents. Mitophagy is a form of selective autophagy during which damaged mitochondria are removed to aid in sequestration of unsalvageable mitochondrial population^16, 18^. During mitophagy, damaged mitochondria are taken up selectively by autophagosomes, which eventually fuse with lysosomes to generate single-membrane autolysosomes that mediate the degradation of the mitochondria^19^. One of the key drivers of mitophagy is sustained depolarization of mitochondria beyond a certain threshold mitochondrial membrane potential (∆Ψm). Mitochondrial depolarization often follows a fission event after which mitochondria can restore an intact potential or remain in sustained depolarization state, which triggers a mitophagy pathway. Loss of ∆Ψm leads to translocation of cytosolic ubiquitin ligase Parkin to mitochondria and activates a cascade of events leading to degradation of mitochondrial fusion protein mitofusin 2 (Mfn2)^20^. Reduction of mitofusin capacity targets the mitochondria to a pre-autophagic pool of fragmented mitochondria which leads to autophagosome formation. Microtubule associated protein 1 light chain 3 (LC3) belongs to the family of orthologs of yeast autophagic proteins Atg8^21^. The LC3 proteins are essential in autophagy involved in the biogenesis and transportation of autophagosomes. They form a double-layer membrane containing phagophore and recruit other proteins, which are involved in the autophagic process^22^. It was shown that upon starvation LC3-I was modified to LC3-II, the membrane bound form. LC3-I is conjugated to phosphatidylethanolamine (PE) to form lipidated LC3-II^23^. LC3-II remains on autophagosomes until after autophagosomal fusion with lysosomes occurs and mitochondria become eliminated by mitophagy. Therefore, LC3 is an autophagosomal membrane marker and is indicative of auto/mitophagy process activation^24, 25^.

Previously, we detected loss of ∆Ψm and concurrent mitochondrial fragmentation, suggesting a possibility of dysregulated mitochondrial quality control system accounting for mitochondrial dysfunction in FECD. However, there are no studies on mitochondrial dynamics in corneal endothelium and its potential role in degeneration of aging endothelial cells affected by FECD. The finding of loss of inner mitochondrial membrane potential in FECD led us to develop an *in vitro* model, which analyzes the direct influence of sustained mitochondrial depolarization with carbonyl cyanide *m*-chlorophenyl hydrazone (CCCP) on mitochondrial dynamics in HCEnCs. Herein, we detected a robust activation of mitophagy pathways accounting for loss of mitochondrial mass in FECD. Furthermore, we detected Mfn2 downregulation, accounting for the loss of fusion capacity in FECD. Moreover, we performed ultrastructural analysis of post-keratoplasty specimens of endothelium, which allowed us, for the first time, to identify extensive autophagic vacuoles containing mitochondrial structures in FECD. Taken together, these findings demonstrate a novel pathogenic mechanism of mitophagy-induced organelle and protein degradation involved in post-mitotic cell loss in ocular tissue.

## Results

### Mitochondrial mass is decreased in FECD

**Figure 1:**
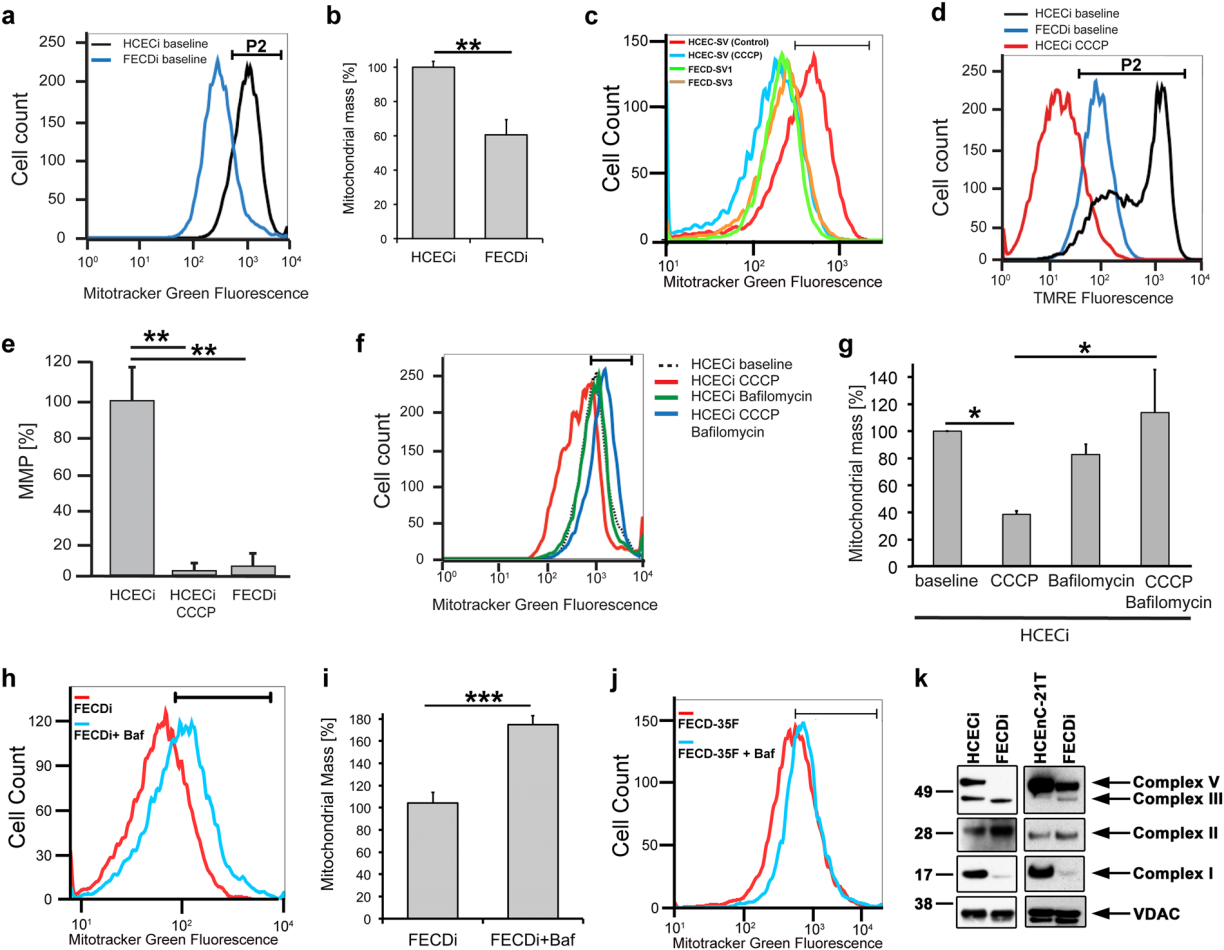
FECD cell lines exhibit decreased mitochondrial membrane potential and mass. (**a**) Representative histogram of Mitotracker Green FM fluorescence intensity plotted against number of cells shows decreased mitochondrial mass in FECDi within the population of cells present in gate P2. (**b**) Quantification of mitochondrial mass from three independent experiments in FECDi. Bars show average percentage of cells in gate P2 (+SEM) from three experiments with HCECi normalized to 100%. (**c**) Two FECD (FECD-SV1 and –SV3) and one normal (HCEC-SV) corneal endothelial cell lines generated with SV40 transduction shows reduced mitochondrial mass in FECD-SV1 and –SV3 that overlaps 20 μM CCCP-treated HCECi-SV. (**d**) Flow cytometry analysis of mitochondrial membrane potential (∆Ψm) measured by TMRE in HCECi and FECDi. HCECi treated with 20 μM CCCP resulted in the abrogation of ∆Ψm in HCECi that is comparable to FECDi. (**e**) Bars represent average percentage of cells in gate P2 (+SEM) from three independent experiments. (**f**,**g**) Flow cytometry analysis and quantification of mitochondrial mass in HCECi treated with 20 μM CCCP, 10 nM bafilomycin, or both with Mitotracker Green FM. Mitochondrial mass in HCECi is reduced with CCCP and is rescued by bafilomycin. (**h**) FECDi cells treated with 10 nM bafilomycin for 16 h shows an increase in mitochondrial mass and quantified from three independent experiments in (**i**). (**j**) Mitochondrial mass is also increased in FECD primary cells (FECD-35F) treated with bafilomycin. (**k**) Western blot of whole cell lysates from two normal (HCECi and HCEnC-21T) and FECDi cell lines shows absence of complex I and V of the electron transport chain in FECDi. VDAC was used as a loading control. Student’s *t*-test was performed to test the statistical significance. *P < 0.05, **P < 0.01, ***P < 0.001.

Since we detected significant mitochondrial fragmentation in FECD *ex vivo* specimens and corneal endothelial cell lines subjected to oxidative stress^13^, we sought to investigate whether mitochondrial mass is affected by the dystrophy. Since one of the hallmark findings in FECD has been mitochondrial depolarization and loss of ATP production, we hypothesized that loss of ∆Ψm leads to activation of mitophagy and a decrease in mitochondrial mass in FECD. We modeled loss of ∆Ψm observed in FECD by treating normal endothelial cells with electron transport chain uncoupler, CCCP. ∆Ψm was determined by flow cytometry employing the potential-sensitive dye TMRE. Using flow cytometry, we compared mitochondrial mass by determining the intensity of MitoTracker Green FM, a dye that accumulates in mitochondria, (Fig. 1a) between two normal endothelial cell lines (HCECi, HCEC-SV) and three diseased cell lines with variable CTG repeat lengths in *TCF4* (FECDi, FECD-SV1, FECD-SV3) (Supplementary Fig. S1). FECDi cells had a significantly lower amount of mitochondrial mass (by 40%, P = 0.0013) compared to HCECi (Fig. 1b). FECD-SV1 (*TCF4* > 40) and FECDSV3 (*TCF4* < 40) showed lower mass than HCEC-SV (33.2% and 46.3% respectively) that overlapped with CCCP-treated HCEC-SV (31.8%, Fig. 1c), indicating that FECD cell lines generated from different donors showed similar results, independent of genetic background. These data were consistent with previous results showing markedly decreased mtDNA copy number and loss of ∆Ψm in FECD^13^. CCCP treatment induced a >90% loss of ∆Ψm in normal cells (P < 0.01) and was similar to FECDi at baseline (P < 0.01) suggesting an abrogation of ∆Ψm in FECDi (Fig. 1d and e).

To determine if mitophagy is activated to account for the loss of mitochondrial mass, bafilomycin, that inhibits the fusion of autophagosomes with lysomes, was used to assess mitochondrial mass with and without the uncoupler. Treatment of HCECi with CCCP decreased mitochondrial mass by 60% (P = 0.02). Bafilomycin did not exhibit a significant effect on mitochondrial mass when used alone, but when co-treated with the uncoupler, it rescued the mitochondrial mass to baseline levels (*P* = 0.012), indicating that mitophagy was upregulated with CCCP (Fig. 1f and g). In addition, bafilomycin also rescued mitochondrial mass in FECDi (by 68%, P = 0.0002) (Fig. 1h and i) as well as in primary FECD cells from a 35 y female donor at passage 3 by 38% (Fig. 1j). These data indicate that disruption of ∆Ψm likely activates mitophagy leading to loss of mitochondrial mass in FECD.

To further assess if an aberrant composition of the electron transport chain (ETC) complexes contributed to the loss of ∆Ψm in FECD, we compared the profile of ETC proteins between two normal CE cell lines (HCECi and HCEnC-21T) and FECDi with a cocktail of antibodies that detects all complexes of the ETC. Figure 1k shows reduced complex I (NADH dehydrogenase) in FECDi, which is one of the three complexes that is involved in pumping protons suggesting that the lack of ∆Ψm is likely due to the loss of complex I. We also detected a complete loss of complex V (ATP synthase) in FECDi that explains reduced ATP levels observed in FECD reported in our recent study^13^.

### Autophagy marker LC3-II to –I ratio and LAMP1 are increased in FECD

**Figure 2:**
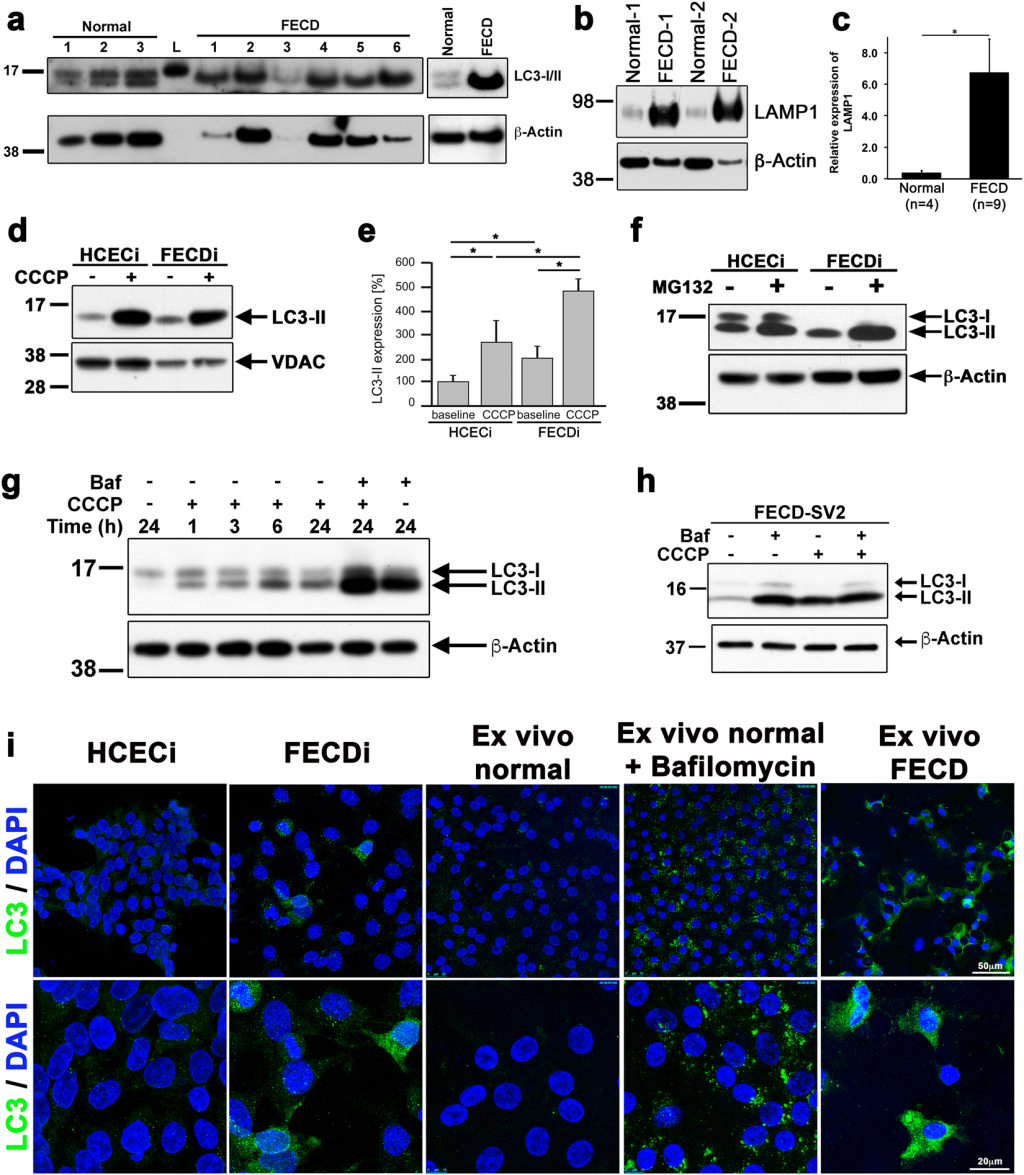
Autophagy marker LC3-II to –I ratio is increased in FECD. (**a**) LC3-I to LC3-II protein expression was compared between normal (n = 3) and FECD (n = 6) specimens. Normal specimens expressed both LC3-I and –II, whereas LC3-I was not detected in FECD specimens. LC3-II was markedly higher in FECD specimens indicating an increased activation of autophagy. (*right*) Western blotting of an independent normal and FECD specimen shows elevated LC3-II levels with the absence of LC3-I. (**b**) Normal and FECD specimens tested for the expression of the lysosomal marker LAMP1 and quantified in (**c**). (**d**) Western blotting of mitochondrial fractions from HCECi and FECDi cells treated with CCCP probed for LC3 shows increased LC3-II in FECDi that is further activated with CCCP. (**e**) Quantification of LC3 in (**d**) normalized with VDAC. Bars represent the average (+SEM) of normalized LC3-II from three independent experiments. (**f**) HCECi and FECDi were exposed to the proteasome inhibitor 50  μM MG132 for 6 hours. LC3-II expression was increased in FECDi compared to HCECi, indicating autophagy pathway is activated after inhibition of the proteasome. (**g**) Treatment of HCEnC-21T cells with 20 μM CCCP shows a time-dependent increase in conversion of LC3-I to LC3-II. Inhibition of the fusion of autophagosome with lysosome with 10 nM bafilomycin leads to the accumulation of LC3-II that is further exacerbated when combined with CCCP. (**h**) Western blot of FECD-SV2 lysates show increased LC3-II that is upregulated with bafilomycin and CCCP. (**i**) Confocal micrographs showing immunolocalization of LC3 in HCECi and FECDi suggests increased LC3 in FECDi. A normal donor cornea was cut into two halves were either untreated or treated with 10 nM bafilomycin for 16 h. Increased LC3 staining was seen in the bafilomycin treated specimen (positive control). FECD *ex vivo* specimen showed increased LC3 with higher intensity compared to the positive control suggesting increased autophagy in FECD. Student’s *t*-test was performed to test the statistical significance. *P < 0.05 and **P < 0.01.

The loss of ∆Ψm and mitochondrial mass in primary FECD CECs as well as FECDi suggested the possibility of increased degradation of mitochondria (mitophagy). LC3-II is a critical component of autophagosomes that is integrated in the membrane following conjugation of the cytosolic LC3-I with phosphotidylethanolamine^26^. Therefore, we analyzed the levels of LC3-II in FECD *ex vivo* specimens as a read out to determine whether loss of mitochondrial mass is caused by enhanced mitophagy/autophagy. The conversion of LC3-I to LC3-II in FECD was compared with normal corneal endothelium (Fig. 2a). FECD specimens showed only the presence of the LC3 isoform associated with the heightened autophagosome content (LC3-II), while normal specimens displayed an equal density of both LC3-I and -II. This result indicates that the autophagy marker LC3-II to -I ratio is upregulated in FECD. Moreover, autolysosome marker lysosomal-associated membrane protein 1 (LAMP1) an ~18-fold (P = 0.018) increase in FECD specimens compared to normal specimens (Fig. 2b and c).

To determine if LC3-II specifically localizes to mitochondria and whether loss of ∆Ψm contributes to the mitophagy observed in FECD specimens, mitochondrial fractions of normal (HCECi) and FECD (FECDi) cell lines were isolated and LC3 protein levels were investigated. At baseline, FECDi showed higher expression of LC3-II compared to normal cells (by 98%; P = 0.04, Fig. 2d and e). This result was further augmented in the presence of mitophagy inducer, CCCP. Expression of LC3-II was 2.75-fold higher in CCCP-treated HCECi cells (P = 0.03) and 4.88-fold higher CCCP-treated FECDi cells (P = 0.001) as compared to untreated, indicating that FECD has heightened susceptibility to mitophagy activation with CCCP (Fig. 2d and e).

Cellular homeostasis is maintained by two main mechanisms such as autophagy and the ubiquitin-proteasome system, both of which are critical for clearing dysfunctional mitochondria^27^. To evaluate the effect of proteasome on activation of mitophagy, HCECi and FECDi cells were treated with the proteasome inhibitor MG132. At baseline LC3-I was undetectable in FECDi whole cell lysates compared to HCECi with the presence of only LC3-II suggesting an upregulated autophagy. The inhibition of proteasome resulted in a modest increase in LC3-II to -I ratio in HCECi but led to a much more dramatic increase in LC3-II to -I ratio in FECDi (Fig. 2f). This suggested that inhibition of proteasomal pathway stimulated autophagy at a greater level in the diseased cells. Furthermore, induction of ∆Ψm loss with CCCP in HCEnC-21T (Fig. 2g) and FECD-SV2 (Fig. 2h) and inhibition of autophagosome formation with bafilomycin led to dramatic LC3-I to –II conversion indicating enhanced autophagosome formation and auto/mitophagy activation in FECD.

To visualize the autophagy activation in cell lines and *ex vivo* corneal endothelium, immunofluorescence studies with LC3 were performed. Normal endothelial cells displayed diffuse LC3 staining (Fig. 2i top panel), while the FECDi showed punctate LC3 staining with increased intensity distributed throughout the cytoplasm at baseline (Fig. 2i bottom panel). Furthermore, immunofluorescence staining of *ex vivo* tissues detected punctate LC3 distribution in the cytoplasm of cells surrounding characteristic rosettes of FECD specimen (73 y, male) (Fig. 2i). Such pattern was absent in normal specimen (56 y, female) but pretreatment with the autophagy inhibitor bafilomycin A showed a similar punctate pattern seen in FECD specimen (Fig. 2i), indicating upregulation of LC3-II after inhibition of autophagic flux.

### Autophagic structures contain mitochondria in FECD

**Figure 3:**
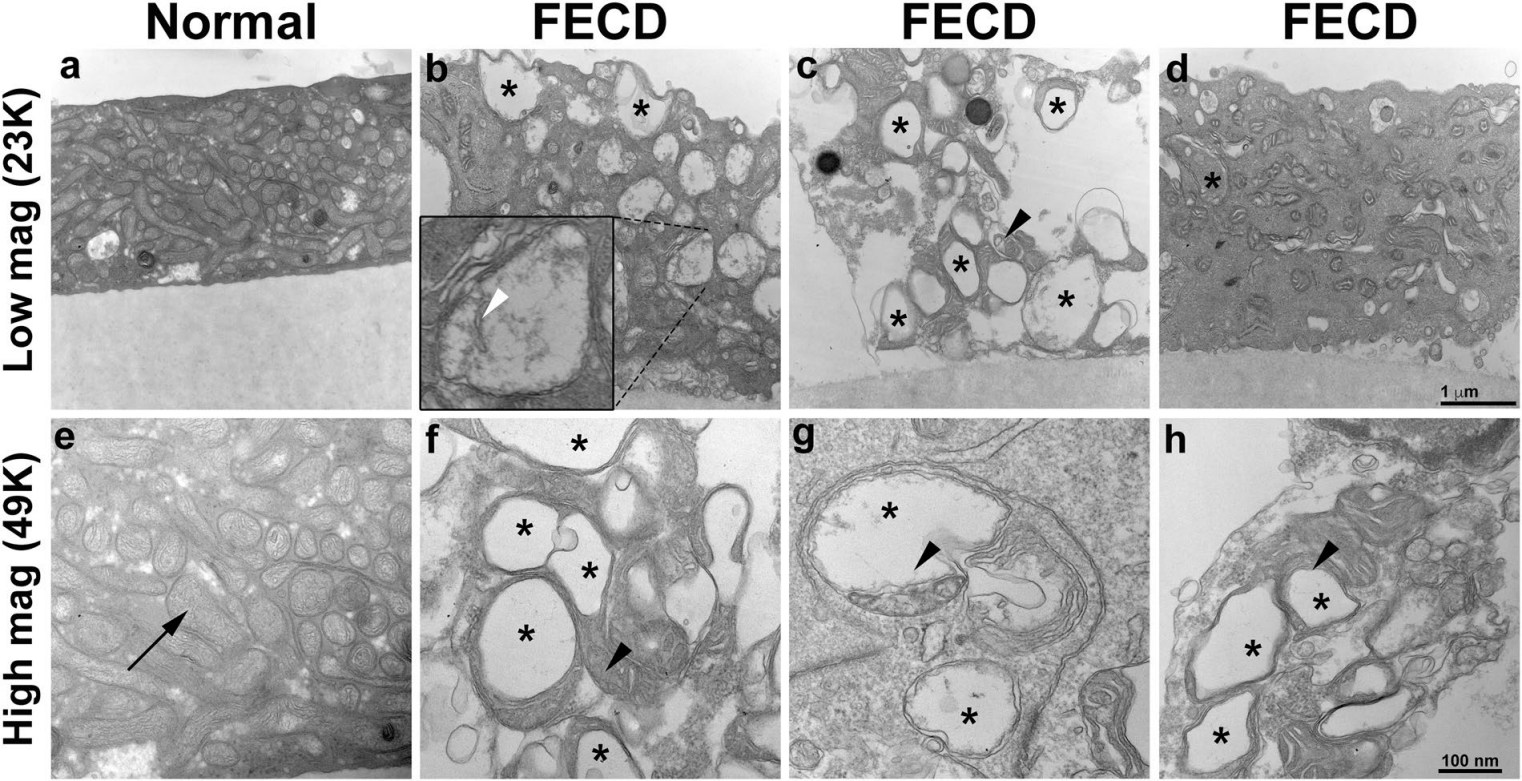
FECD specimens reveal an abundance of autophagic structures and abnormal mitochondria with loss of ETC complex. Transmission electron micrographs of a normal *ex vivo* specimen (**a**,**e**) show the presence of high density of normal mitochondria (black arrow) in a corneal endothelial cell. Panels b–d and f–h are representative images of FECD *ex vivo* specimen that suggest increased number of autophagic structures in the form of vacuoles (black stars). Black arrowheads in panels **c**,**f**–**h** indicate autophagosomes containing mitochondria and white arrowhead in panel ii indicates degraded mitochondrial cristae.

Transmission electron microscopy (TEM) is a standard method for monitoring cellular ultrastructure and autophagy/mitophagy^28^. TEM revealed a decrease in total number of mitochondria in FECD *ex vivo* specimens as compared with controls (n = 2). FECD specimens (n = 3) displayed an apparent increase in the number of vacuoles or vesicular-like structures in the cytoplasm, indicating a greater increase in autophagic structures in FECD (Fig. 3). Some autophagic vacuoles contained mitochondria in them (Fig. 3 black arrowhead). In addition, severely swollen and disrupted mitochondria were seen in FECD compared to normal cornea (Fig. 3, white arrowhead). Moreover, partial or complete cristolysis was the most commonly altered feature observed in FECD.

### Mitophagy drives loss of mitochondrial fusion protein Mfn2 in FECD

**Figure 4:**
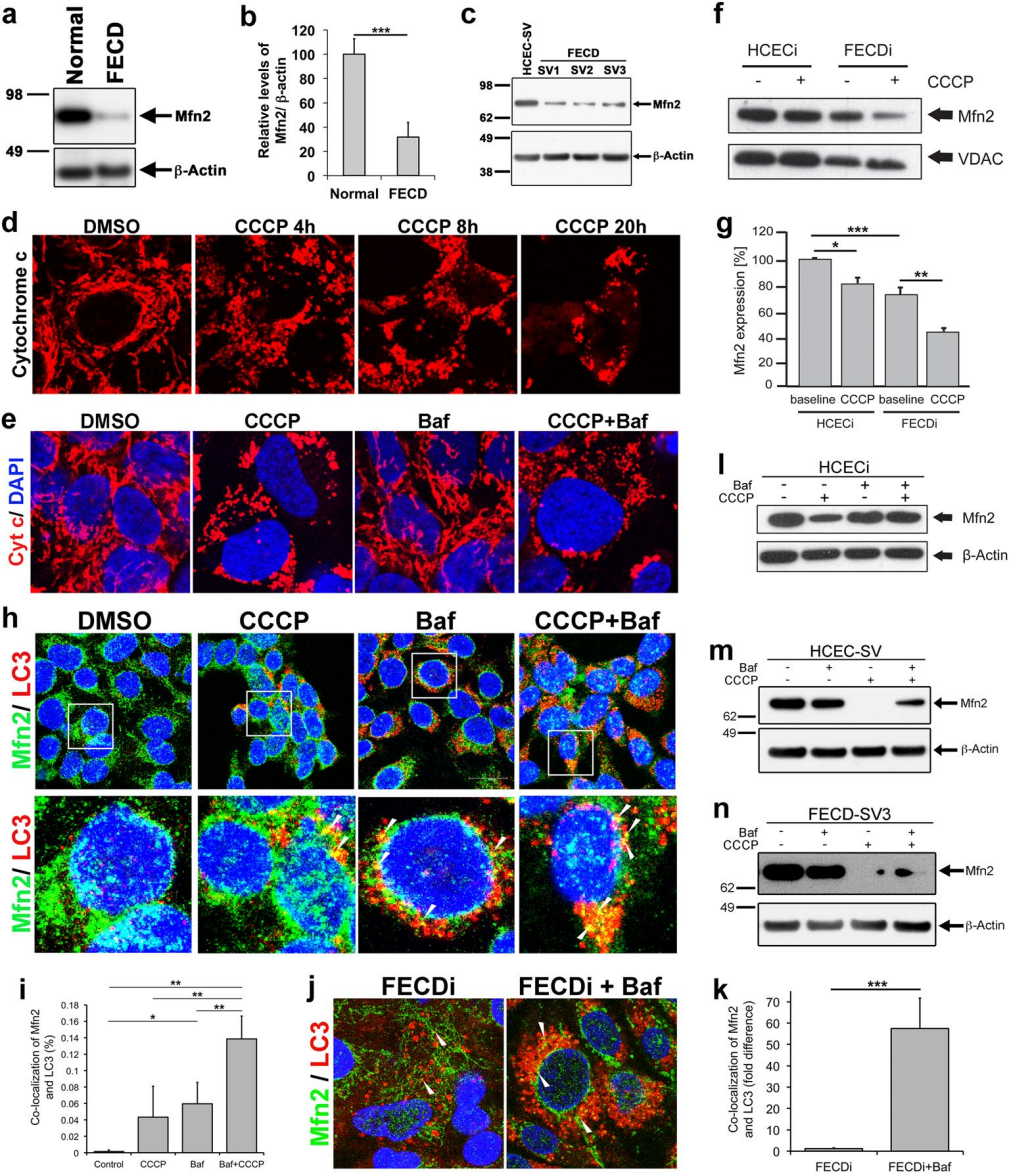
Mitophagy drives loss of mitochondrial fusion protein Mfn2 in FECD. (**a**) Representative western blot of Mfn2 shows reduced expression in an FECD specimen compared to normal donor. (**b**) Quantification of western blot of Mfn2 (average + SEM) normalized with β-actin from normal donors (n = 10) and FECD *ex vivo* specimens (n = 10) showed a loss of Mfn2 in FECD (*P* = 0.0005, Student’s *t*-test). (**c**) Western blot of SV40 immortalized normal (HCEC-SV) and FECD cell lines (FECD-SV1, -SV2, and -SV3) show reduced Mfn2 levels that corroborated with human specimens. (**d**) Time course of mitochondrial fragmentation observed with the immunolocalization of cytochrome *c* in normal corneal endothelial cell lines treated with 20 μM CCCP. (**e**) CCCP- induced mitochondrial fragmentation is unaltered with autophagosome inhibitor bafilomycin (10 nM) (CCCP + baf panel), whereas bafilomycin alone does not disrupt the normal mitochondrial architecture (Baf panel). (**f**) Western blot shows a reduction of Mfn2 in the mitochondrial fractions in normal cell line treated with CCCP as well as basal levels in FECDi cells. (**g**) Quantification of Mfn2 expression levels normalized with VDAC representing average + SEM of three independent experiments. CCCP induces a 42% reduction in Mfn2 in FECDi cells compared to a 20% decrease in HCECi. (**h**) Mfn2 co-localizes with LC3 that visualized moderately in normal cells treated with 20 μM CCCP for 8 h and increased spots of co-localization are observed with 10 nM bafilomycin (white arrowheads) suggesting degradation of Mfn2 through mitophagy. (**i**) Quantification of co-localization of LC3 and Mfn2 shows an increase in co-localization in bafilomycin treated HCECi cells and a further increase in cells treated with CCCP and bafilomycin. (**j**) FECDi cells show increased autophagosome formation visualized with LC3 staining that appear as vesicles (left panel). Treatment with 10 nM bafilomycin for 16 h led to increase in colocalization of Mfn2 and LC3 (right panel). (**k**) Quantification of co-localization of Mfn2 and LC3 in FECDi cells shows an increase in cells treated with bafilomycin. (**l**–**n**) Treatment with CCCP and bafilomycin stabilized Mfn2 protein levels as compared to CCCP treatment alone in two normal (HCECi, panel j; HCEC-SV, panel k) and one FECD cell line (FECD-SV3, panel l) suggesting that activated mito/autophagy degradation pathway likely accounts for Mfn2 protein degradation in FECD. Student’s *t*-test was performed in (**b**,**k**) and one-way ANOVA in (**i**) to test the statistical significance. *P < 0.05, **P < 0.01 and ***P < 0.001.

Next, we sought to investigate whether heightened auto/mitophagy activation was related to deficient mitochondrial quality control. Based on functional relevance of mitochondrial fusion protein mitofusin 2 (Mfn2) in aging and ROS-dependent signaling^29^, we analyzed the role of Mfn2 in triggering an abnormal mitophagy in FECD. Mitofusin 2 (Mfn2) is essential for the fusion of mitochondria and is involved in stabilizing the interaction between the two mitochondria, thus preventing stress-induced fragmentation and mitophagic clearance^30^. We detected a marked reduction of Mfn2 in FECD as compared to normal specimens (n = 10, P = 0.0005, Fig. 4a and b). A similar decrease in Mfn2 was also observed consistently in recently generated FECD cell lines (-SV1, -SV2, and –SV3), irrespective of differences in *TCF4* repeat expansion (Fig. 4c).

Reduction of Mfn2 in FECD was not caused by decreased gene expression (Supplementary Fig. S2), indicating that an altered protein turnover is likely to account for reduced Mfn2 expression in diseased cells. To gain insight into the etiology of Mfn2 loss, we modeled low-∆Ψm -induced mitochondrial fragmentation detected in FECD^13^ by treating endothelial cells with ∆Ψm uncoupler CCCP. Treatment with CCCP induced a transition from a typical tubular mitochondrial network into punctiform mitochondria, indicating time-dependent increase in mitochondrial fragmentation (Fig. 4d). These findings mimicked mitochondrial fragmentation of FECD *ex vivo* specimens^13^. Addition of bafilomycin alone did not alter mitochondrial morphology. Moreover, addition of bafilomycin to CCCP did not rescue CCCP-induced mitochondrial fragmentation, suggesting that the inhibition of autophagy had little effect on the mitochondrial morphology (Fig. 4e).

Mfn2 is involved in outer mitochondrial membrane fusion and is a mitochondrial membrane associated protein. Therefore, a highly pure mitochondrial fraction was isolated using anti-TOM22 conjugated magnetic beads from untreated or CCCP-treated HCECi and FECDi cells and probed for Mfn2. At baseline, FECDi exhibited lower mitochondrial Mfn2 levels by 27% (P = 0.007) as compared to normal cells. Induction of mitochondrial fragmentation with CCCP further diminished mitochondrial Mfn2 in normal cells by 20%, (P = 0.02). Treatment of FECDi with CCCP resulted in a 46% decrease compared to baseline FECDi cells (P = 0.007) (Fig. 4f and g).

It is known that loss of ∆Ψm triggers ubiquitination of mitochondrial proteins, such as Mfn2, and leads to its degradation, uncounteracted fission, and subsequent mitophagy^31^. To investigate whether Mfn2 decrease was due to enhanced ubiquitination, immunoprecipitation of Mfn2 was performed using Mfn2-specific antibody and anti-ubiquitin western blotting was performed. We did not detect enhanced Mfn2 ubiquitination in either HCECi or FECDi cells, indicating that proteasomal influence is unlikely the cause of Mfn2 degradation in FECD (Supplementary Fig. S3a). Next, we induced loss of ∆Ψm with CCCP and menadione (MN)^13^ and detected that proteasomal inhibitors, MG132 and epoxomicin^32^, did not rescue Mfn2 levels in either HCECi or FECDi (Supplementary Fig. S3b,c). These results indicate that during stress, stabilization of ubiquitin-proteasome pathway did not increase Mfn2 levels in whole cell lysate nor purified mitochondria; thus, an alternate pathway might be involved in Mfn2 degradation.

To investigate a possible role of mitophagy in Mfn2 turnover, we examined colocalization of Mfn2 and LC3 during the process of mitochondrial fragmentation (Fig. 4h).

Immunocytochemical localization of LC3-II showed increased puncta in both CCCP and bafilomycin-treated cells (Fig. 4h). The intensity of the LC3 staining increased in cells treated with a combination of CCCP and bafilomycin. The staining of Mfn2 localized to the mitochondria and exhibited a partial co-localization of Mfn2 and LC3 (Fig. 4h arrowheads) in cells treated with CCCP and bafilomycin independently. This co-localization increased in cells treated with CCCP and bafilomycin together (Fig. 4h arrowheads). Quantitative analysis of immunofluorescence detected an increase in co-localization percentage with bafilomycin and CCCP treated cells as compared to controls (P = 0.001), or CCCP alone (P = 0.0012), and bafilomycin alone (P = 0.0026) treatments (Fig. 4i, Supplementary Fig. S4). In addition, Mfn2 co-localized with LC3 in FECDi under basal conditions and treatment with bafilomycin showed a marked increase in the co-localization of Mfn2 and LC3 in FECDi (Fig. 4j and k, P = 5.24 × 10^−5^), indicating that inhibition of auto/mitophagy rescues degradation of Mfn2. To corroborate this, we found that treatment with CCCP and bafilomycin stabilized and upregulated Mfn2 protein levels as compared to CCCP treatment alone in HCECi (Fig. 4l) and HCEC-SV (Fig. 4m). Moreover, even in FECD-SV2, Mfn2 levels were rescued after mitophagy inhibition (Fig. 4n). These data suggest that activated mito/autophagy degradation pathway likely accounts for Mfn2 protein degradation in FECD.

## Discussion

Mitochondrial dysfunction is virtually at the core of all neurodegenerative disorders and connected to the aging process^33–36^. Keeping mitochondria in a healthy state is a complex process and has to be tightly regulated via mitochondrial quality control mechanisms and complex interplay between mitochondrial biogenesis and degradation^37, 38^. FECD is an oxidative stress disorder of the mitotically incompetent corneal endothelial cells, which are highly dependent on mitochondrial quality control mechanisms to maintain mitochondrial integrity and cellular livelihood^9^. Our study provides first line of evidence supporting the involvement of mitophagy in pathogenesis of FECD. Previous studies detected that heightened mitochondrial DNA damage^39^, loss of mitochondrial membrane potential, and decreased ATP production were associated with mitochondrial fragmentation, pointing to dystrophy-induced mitochondrial dysfunction^13^. Our data indicate that downstream induction of auto/mitophagy leads to a decrease in mitochondrial mass and depletion of functional mitochondria, accounting for perturbation of bioenergetics of the cell seen in FECD^14, 40^. Interestingly, by blocking autophagic flux, we were able to rescue mitochondrial mass in FECD. Ultrastructural studies of FECD tissues revealed striking presence of autophagic structures with degenerated and internalized mitochondria, indicating excessive engulfment of mitochondria by autophagic vesicles. Moreover, we identified an increase in autophagosmes and autopagolysosomes in FECD, indicating the upregulation of the autophagolysosomal system. A concurrent decline in mitochondrial Mfn2, which is key fusion protein targeting the salvageable mitochondria into functional networks, was indicative of lost fusion capacity and subsequent activation of fission-induced mitophagy. Future studies are needed to determine whether decline in lysosomal degradation plays a role in the abnormal clearance of autophagic structures in FECD.

The evidence for loss of mitochondrial biogenesis in FECD was further supported by decline in the levels of electron transport chain subunits, complexes I and V, the loss of which led to sustained mitochondrial depolarization^13^, likely further reducing Mfn2 capacity and inciting the autophagic machinery. Interestingly, we detected that loss of Mfn2 was unexpectedly driven by auto/mitophagy. We showed that Mfn2 was incorporated in the autophagosome formation pointing to the novel pathway of Mfn2 protein turnover via auto/mitophagic degradation. Previous studies have shown that Mfn2 is a target of proteasomal degradation^41–44^. However, we did not detect heightened Mfn2 ubiquitination and proteasomal degradation in FECD. In corneal endothelial cells, inhibition of the proteasome with MG132 and epoxomicin did not recover the levels of Mfn2, while the inhibition of the autophagy with bafilomycin stabilized Mfn2 levels and co-localized it to the mitochondrial autophagosomes stained with LC3. These results indicated that Mfn2 degradation was likely not dependent on ubiquitin-proteasome pathway. The difference in our results from previous reports that show ubiquitin-proteasome-dependent Mfn2 degradation may be due to the use of Parkin-transfected cell lines^45–47^. Since our preliminary studies have detected adequate levels of endogenous Parkin in normal corneal endothelium and altered levels in FECD^48^, we did not overexpress Parkin in order to retain endogenous differences seen in the disease. Future studies are needed to evaluate the role of Parkin in mitochondrial biogenesis relative to Mfn2 degradation and activation of mitophagy seen in corneal cells.

Loss of Mfn2 in FECD, which is an age-related disorder of corneal endothelium, is consistent with previous reports showing a decline in Mfn2 in accelerated aging of muscle cells^29^ and in Alzheimer’s disease (AD) models^49^. In AD, amyloid-beta plaque deposition reduced Mfn2 levels and induced mitochondrial fragmentation leading to antioxidant Peroxiredoxin 2 (Prx2) inactivation. Similarly to AD, extracellular matrix deposits in the form of guttae are interspersed among cells exhibiting mitochondrial fragmentation in FECD. Moreover, FECD cells show loss of peroxiredoxins, mainly Prx2, leading to perturbation of oxidant-antioxidant balance^9, 12^. Interestingly, Mfn2 has been linked to the maintenance of mitochondrial electrochemical gradient^50^ and as an anti-apoptotic factor^51, 52^. We have observed a significant compromise of both components in FECD^5, 7, 8, 13^.

The main question that arises is whether activation of mitophagy is beneficial to the endothelial cell livelihood, by way of adapting it to stress, or detrimental, by destroying the ‘powerhouses’ of the cell and destining the cell to eventual apoptosis^53^. Apoptosis has been the predominant mechanism involved in endothelial cell loss in FECD via p53-dependent and caspase-3 and -9-dependent mechanisms in the oxidative stress models and native tissue specimens^5, 8, 54, 55^. Specifically, activation of intrinsic apoptotic pathway was shown to be initiated by DNA damage, which culminated in mitochondrial permeabilization and release of cytochrome *c* in FECD^13^. Although the interplay between autophagy and apoptosis has been extensively studied in carcinogenesis^56–59^, the purported dual role of autophagy in mediating cell death and cytoprotection in degenerative disorders needs further investigation^60^. The initiation of auto/mitophagy in FECD is likely designed to remove cytoplasmic constituents including damaged mitochondria and misfolded proteins to preserve cellular bioenergetic demands and to recycle the degraded biomolecules^55^. The chronic cellular stress and eventual over-accumulation of autophagosomes may overpower the autophagic machinery resulting in conditions of ‘extreme stress’ that leads to activation of caspase-dependent apoptosis in FECD^61^. Further studies are needed to investigate whether accumulation of autophagic vacuoles induce local production of guttae, the hallmark ‘plaques’ of degenerating corneal endothelium^62^.

## Material and Methods

### Human Corneal Endothelial Cell Culture

Normal (HCECi^63^), and FECD (FECDi^63, 64^) cell lines were gifts from M. Griffith (Ottawa Hospital Research Institute) and R. Mohan (University of Missouri Health System), respectively. We have generated HCEnC-21T^65^ and SV40 T antigen (ALSTEM, Richmond, CA) immortalized cell lines; the latter were derived from normal 67 M donor (HCEC-SV) and three FECD specimens (73 y female, FECD-SV1; 61 y female, FECD-SV2; and 76 y male, FECD-SV3,) at passage 2^66^. Genomic DNA was isolated using Qiagen Blood & Tissue kit (Valencia, CA) and CTG repeat length in *TCF4* of 40 and above were considered expanded^67^. FECD-SV1 harbored a monoallelic expansion, whereas both alleles of *TCF4* were below 40 repeats in FECD-SV2 and -SV3 (Supplementary Fig. S1).

### Human tissue

This study was conducted according to the tenets of the Helsinki Declaration of 1975, as revised in 1983, and approved by the Massachusetts Eye and Ear Institutional Review Board. Written and informed consent was obtained from patients as reported previously^13^. Normal donor corneas were purchased from SightLife (Bethlehem, PA). A total of 23 normal donor corneas (65.7 ± 6.7 y) and 33 FECD (67.5 ± 8.0 y) specimens were used in this study.

### Detection of ∆Ψm and mitochondrial mass

Cells were treated with either 10 nM tetramethylrhodamine ethyl ester (TMRE; Invitrogen) for 30 minutes at 37 °C for ∆Ψm or 50 nM MitoTracker Green FM (Invitrogen) for 30 minutes at 37 °C for mitochondrial mass. Cells were harvested in 0.5% BSA, 50 μM EDTA, in PBS and 10,000 live cells were analyzed by BD LSR II flow cytometer. The live gate was applied to the FITC histogram according to the gate P2 shown in Fig. 1a. The percentage of cells in this gate was analyzed in each sample. The analysis to detect TMRE fluorescence was performed as described for Mitotracker Green.

### Western Blot Analysis

Mitochondria were purified using mitochondria isolation kit (Miltenyi Biotec, San Diego, CA) according to manufacturer’s instructions. Western blotting was carried out as reported previously^13^. Primary antibodies (β-Actin, #A1978, Sigma; Mfn2, ab56889, abcam; LC3A/B #4108, Cell Signaling, OxPhos complex monoclonal antibody (cocktail), 4-8099, Thermo Fisher Scientific; VDAC, ab18988, abcam; LAMP1, 9091, Cell Signaling) were incubated overnight. Relative protein intensity was quantified from scanned films with ImageJ software.

### Immunofluorescence staining

Immunofluorescence staining of cell lines and human specimens were performed as described previously^13^. Primary antibodies for LC-3B (D11) XP rabbit mAb (cat no: 3868, Cell Signaling, Danvers, MA), cytochrome *c* (cat no: 556432, BD Biosciences), and Mfn2 (ab56889, abcam) were incubated overnight at 4 °C. Mfn2 and LC3 co-localization quantification was carried out using the ‘Co-localization Colormap’ plugin for ImageJ^68^. The number of highly co-localized pixels (≥0.5 nMDP) was used to derive percentage co-localization^69^.

### Transmission electron microscopy

Human specimens were fixed in 1/2 strength Karnovsky’s fixative and processed for TEM using standard procedures, as described previously^70, 71^. Briefly, specimens were rinsed in cacodylate buffer, post-fixed in 2% osmium tetroxide, *en bloc* stained with 2% uranyl acetate, dehydrated with alcohol to propelene oxide, and infiltrated with tEPON-812 epoxy resin (Tousimis, Rockville, MD) utilizing an automated EMS Lynx 2 EM tissue processor (Electron Microscopy Sciences, Hatfield, PA). Ultrathin sections (80 nm) were cut from the epoxy block using a Leica EM UC7 ultramicrotome (Leica Microsystems, Buffalo Grove, IL) and a diamond knife. Sections were imaged using an FEI Tecnai G2 Spirit transmission electron microscope (FEI, Hillsboro, Oregon) at 80 kV interfaced with an AMT XR41 digital CCD camera (Advanced Microscopy Techniques, Woburn, MA).

### Immunoprecipitation

HCECi and FECDi cells were lysed with 25 mM Hepes (pH 7.5), 10 mM CaCl_2_, 1% digitonin, 20 mM iodoacetamide, 20mM N-ethylmaleimide, and protease inhibitor cocktail for 30 min on ice. Cleared lysates were mixed with either 2 μg of anti-Mfn2 or isotype control IgG for 2 hours at 4 °C and immunoprecipitated with 50 μl of protein A/G sepharose beads for 2 hours at 4 °C.

### Real-time reverse transcription–polymerase chain reaction

Total RNA was extracted from corneal human specimens (4 FECD and 4 normal corneas) with RNeasy micro kit (Qiagen). 30ng of RNA was used for cDNA synthesis (iScript cDNA synthesis kit, Biorad). Relative expression of MFN2 (Hs00208382_m1) was obtained by normalizing with B2M (cat no: 4333766F) amplified using Probe Fast master mix (Kapa Biosystems).

## Electronic supplementary material

Supplementary Figures
