# Supplementary material for: Activation of mitophagy leads to decline in Mfn2 and loss of mitochondrial mass in Fuchs endothelial corneal dystrophy
Source: Sci Rep. 2017 Jul 27;7:6656. doi: 10.1038/s41598-017-06523-2 (PMC5532298; doi:10.1038/s41598-017-06523-2)
Supplement: Supplementary file 1 — Supplementary Figures [file 41598_2017_6523_MOESM1_ESM.pdf]

Supplementary Figures

Activation of mitophagy leads to decline in Mfn2 and loss of mitochondrial mass in Fuchs endothelial corneal dystrophy

Anne-Sophie Benischke[1], Shivakumar Vasanth[1], Takashi Miyai[1], Kishore Reddy Katikireddy[1], Tomas White[1], Yuming Chen[1], Adna Halilovic[1], Marianne Price[2], Francis Price Jr.[2], Paloma B. Liton[3], Ula V. Jurkunas[1*]

Supplemental Figure 1

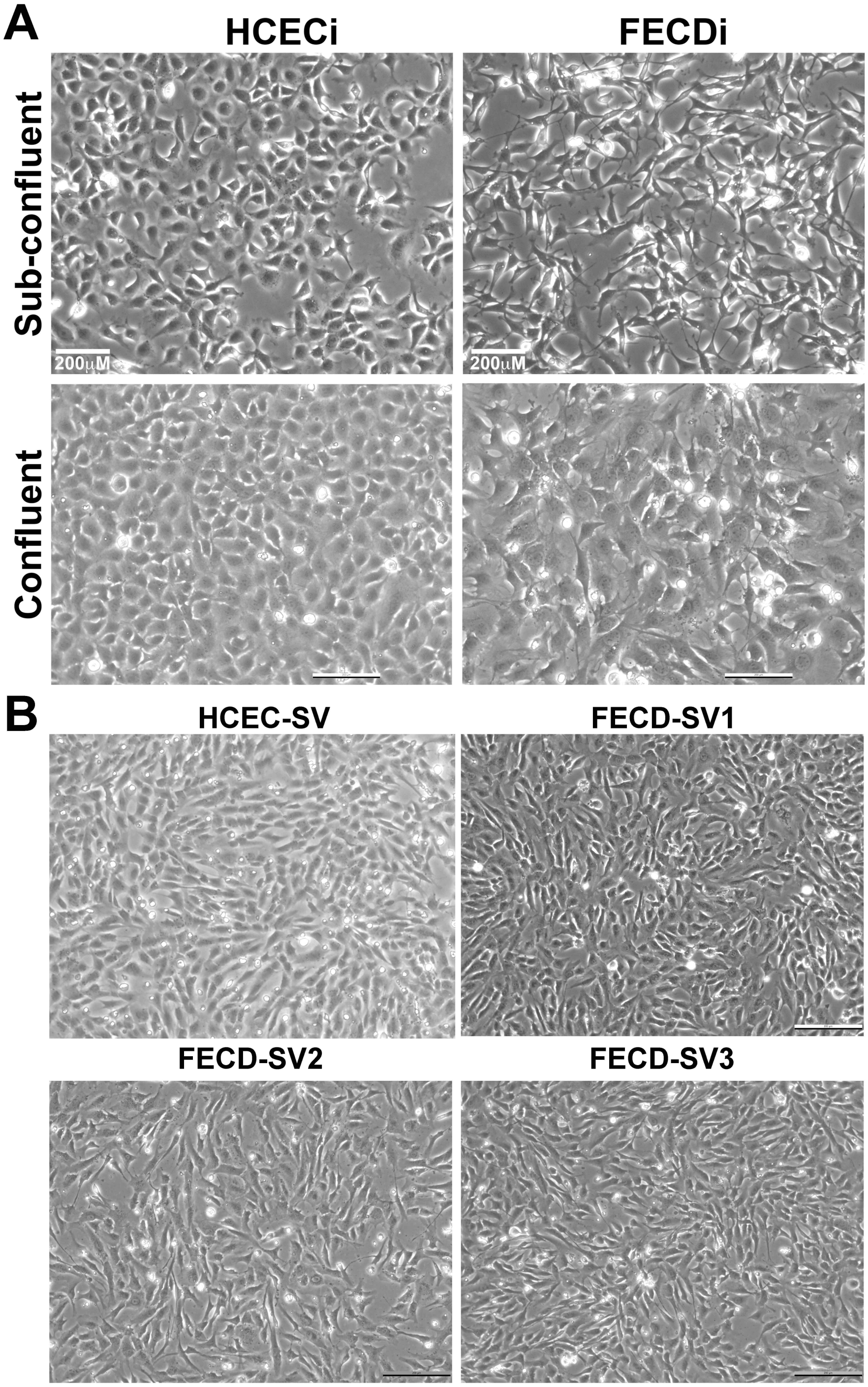

Figure S1. Cellular morphology of immortalized corneal endothelial cell lines. (A) Phase-contrast images of HCECi and FECDi acquired under confluent and sub-confluent conditions suggests an elongated fibroblast-like morphology of FECDi whereas HCECi cells are smaller in shape and show distinct cell boundaries suggestive of contact inhibition. (B) Phase-contrast images of SV40 immortalized cell lines from normal and FECD specimens used in this study.

Supplemental Figure 2

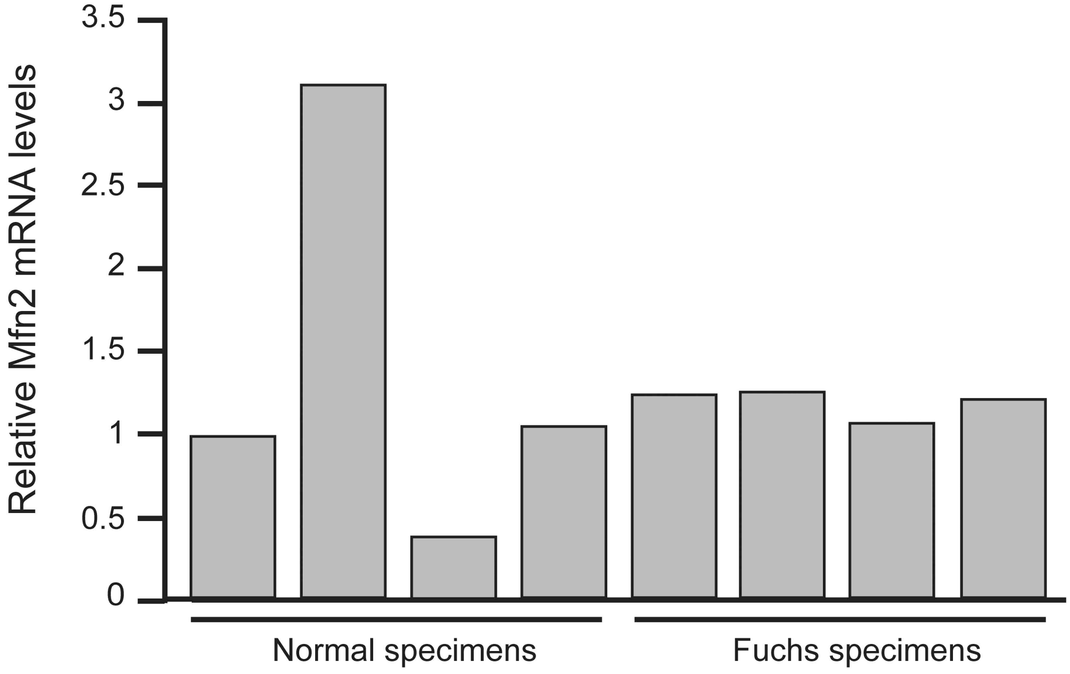

Figure S2. Differential gene expression of Mfn2 in normal and FECD ex vivo specimens. RNA extracted from normal donor CE (n = 4) and FECD (n = 4) specimens were subjected to cDNA synthesis and real-time PCR based gene expression analysis for Mfn2 normalized with b-2 microglobulin (*B2M*). Relative Mfn2 expression does not show a significant change between normal and FECD specimens suggesting the lower protein level of Mfn2 in FECD specimens is not due to changes in transcriptional activity.

Supplemental Figure 3

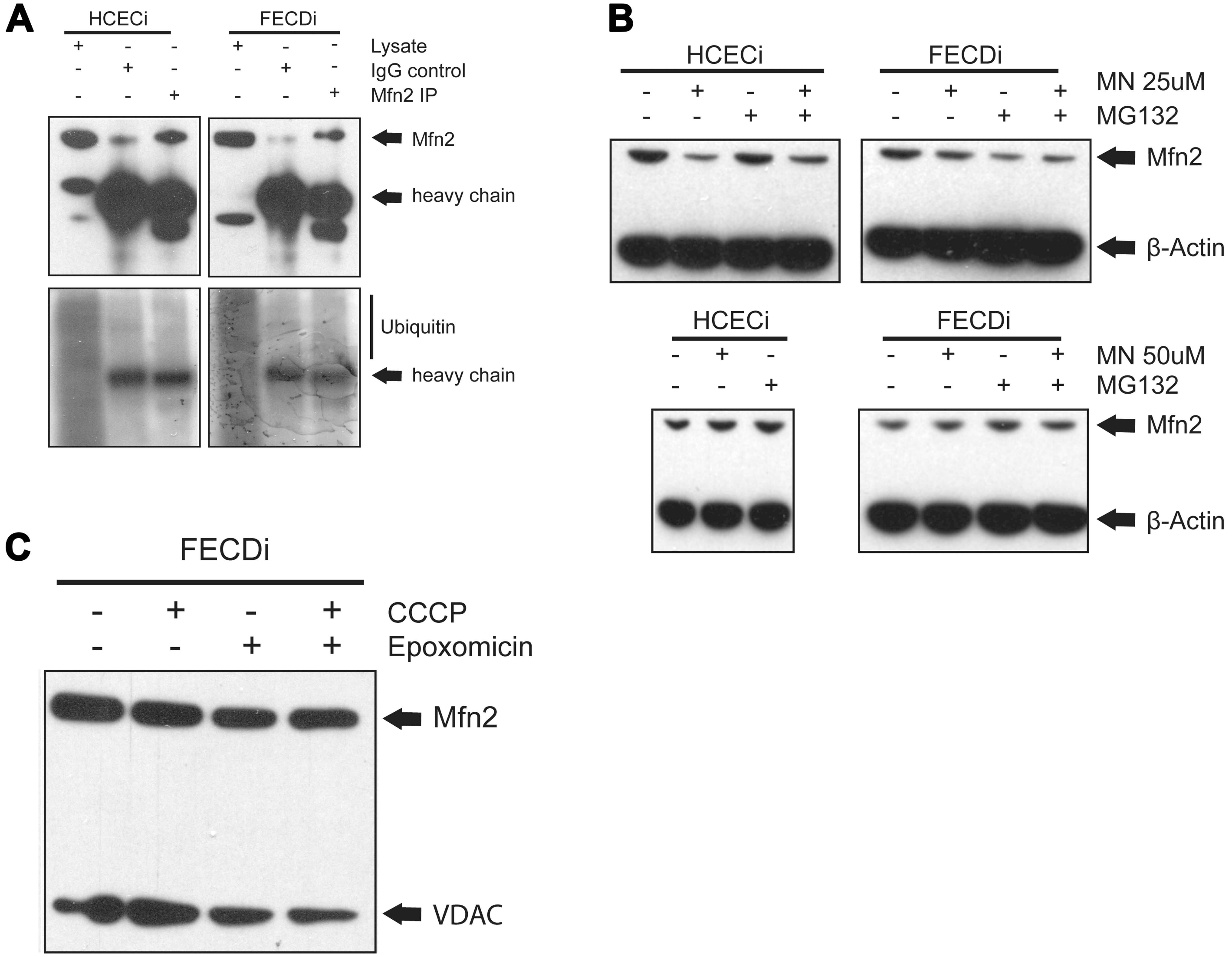

Figure S3. Loss of Mfn2 due to mitochondrial depolarization is not regulated by ubiquitin-proteasome mediated degradation. (**a**) Lysates from HCECi and FECDi were immunoprecipitated with anti-Mfn2 antibody and probed for the presence of ubiquitin. Bottom panel shows the presence of heavy chain cross reacting with the primary antibody and does not reveal any immunoreactivity with anti-ubiquitin suggesting a lack of ubiquitination of Mfn2 in both normal and FECDi cells. (**b**) HCECi and FECDi cells treated with 25mM (top) and 50mM (bottom) menadione that induces oxidative stress results in reduced levels of Mfn2. Treatment with the proteasome inhibitor MG132 does not result in the accumulation of Mfn2. (**c**) Western blotting of mitochondrial fractions suggest degradation of Mfn2 induced by CCCP is not rescued by a selective proteasome inhibitor epoxomicin in FECDi.

Supplemental Figure 4

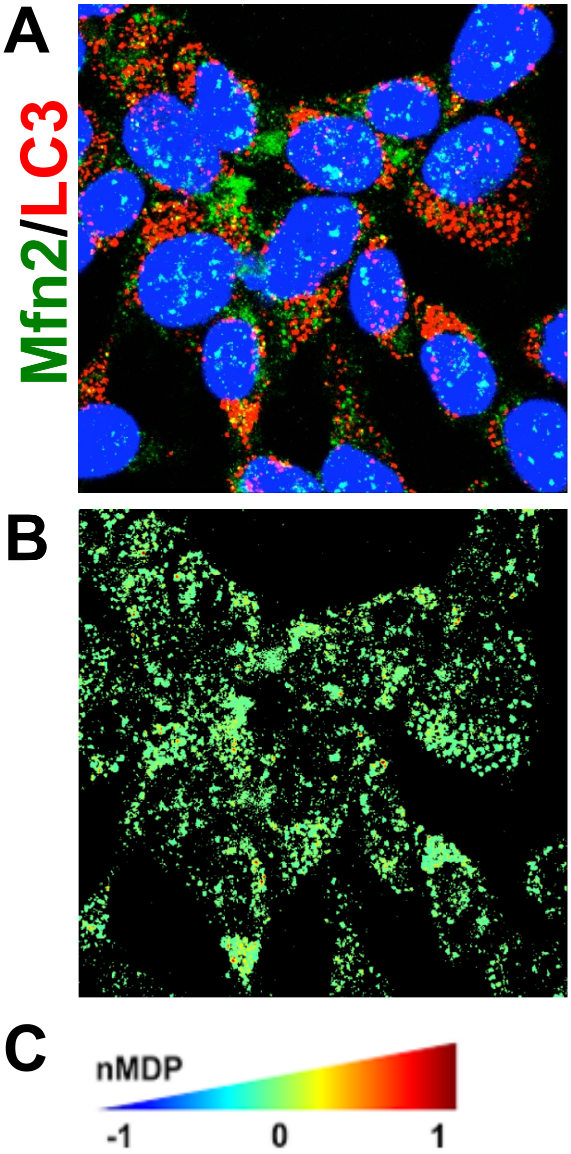

D

nMDP value	count
-1	0
-0.9	0
-0.8	0
-0.7	0
-0.6	0
-0.5	0
-0.4	0
-0.3	0
-0.2	4099
-0.1	200700
0.1	427815
0.2	3072
0.3	865
0.4	376
0.5	232
0.6	141
0.7	82
0.8	72
0.9	53
1	110
total pixels	637617
≥ 0.5 nMDP	690
Co-localization (%)	0.108

Figure S4. Colocalization analysis of Mfn2 and LC3. An example of colocalization analysis is described here using a confocal micrograph acquired from HCEnC-21T cells treated with CCCP and Bafilomycin as shown in (A) as an example. Merged confocal images were split to remove the blue channel (DAPI) before the ‘co-localization colormap’ plugin for Image J was used to visualize the co-localization of red and green fluorescent signals (B). nMDP values range from -1 to 1, where indexes above 0 are represented by hot colors (co-localization) (C). The number of pixels exhibiting strong co-localization, deemed to be nMPD ≥ 0.5, were summed and used with total pixel number to calculate percentage co-localization (D).
